# Seroprevalence of West Nile virus, Greece, 2020

**DOI:** 10.2807/1560-7917.ES.2025.30.15.2400487

**Published:** 2025-04-17

**Authors:** Michalis Koureas, Asimina Nasika, Athanasios G Lianos, Alexandros Vontas, Maria A Kyritsi, Ioanna Voulgaridi, Alexia Matziri, Zacharoula Bogogiannidou, Fani Kalala, Varvara A Mouchtouri, Matthaios Speletas, Christos Hadjichristodoulou

**Affiliations:** 1 Laboratory of Hygiene and Epidemiology, Faculty of Medicine, University of Thessaly, Larissa, Greece; 2 Department of Immunology and Histocompatibility, Faculty of Medicine, University of Thessaly, Larissa, Greece

**Keywords:** West Nile Virus, Seroprevalence, IgG antibodies

## Abstract

**Background:**

West Nile virus (WNV) is a growing public health concern in Europe. Greece is one of the most affected countries in Europe, with the highest annual incidences.

**Aim:**

We aimed at assessing IgG antibodies to WNV in the Greek population and compared the results with a nationwide survey conducted in the period 2012–2013.

**Methods:**

In a geographically stratified sampling, 4,416 serum samples were collected and analysed for WNV-specific IgG antibodies using ELISA. Samples positive for WNV IgG were further tested with a WNV serum neutralisation test to detect false positives.

**Results:**

The weighted seroprevalence, adjusted for age, sex and region, was 2.83% (95% confidence interval (CI): 2.32–3.44) in the 4,416 samples tested, significantly higher than in the 2012–2013 survey (1.55%; 95% CI: 1.17–2.04). The seropositivity increased with age with the highest seroprevalence in persons aged ≥ 80 years (6.04%; 95% CI: 3.28–10.88). No significant differences in seropositivity were observed between sexes or regions. We estimated that 312 (95% CI: 256–379) persons had a WNV infection per a case of West Nile neuroinvasive disease (WNND). A certain degree of discordance was observed between areas with increased seroprevalence and those with an increased incidence of WNND.

**Conclusion:**

Our study reveals a wider geographical spread of WNV infections in Greece compared with previous investigations. The nearly twofold increase in seroprevalence highlights the need for ongoing monitoring and preventive measures to mitigate the impact of WNV on public health in Greece.

**Key public health message**

**What did you want to address in this study and why?**

West Nile virus (WNV) is transmitted to humans and other animals via mosquitoes of the genus *Culex*. Infection with the virus can lead to a severe neurological disease. In 2020, we collected blood samples from Greece, where WNV was first detected in 2010, and tested them for antibodies against WNV indicating a previous infection and compared the results with a previous study conducted 2012–2013.

**What have we learnt from this study?**

Of the 4,416 serum samples tested, 2.83% had antibodies against WNV, more than the 1.55% in the previous survey. The virus is spread throughout the country. Adults aged 80 years and older were more often seropositive, whereas no significant differences were observed between males and females or regions. We estimated that 312 persons had a WNV infection per patient diagnosed with severe disease.

**What are the implications of your findings for public health?**

The observed increase in the exposure to WNV underlines the need for increased awareness and strengthened surveillance. As older adults are more susceptible to a severe disease, targeted preventive measures, such as mosquito control, education on reducing breeding sites, and personal protection, are necessary.

## Introduction

West Nile virus (WNV) is a positive-stranded RNA virus in the family of Flaviviridae (genus Flavivirus), which includes pathogens such as dengue virus, yellow fever virus and Japanese encephalitis virus [1]. Avian species are considered the primary hosts of WNV, and in an endemic region, the virus is maintained in an enzootic cycle between mosquitoes and birds [2]. A wide variety of bird species have been implicated, with those in the Passeriformes order (including the Corvidae, Fringillidae and Passeridae families) and the Charadriiformes order (specifically the Laridae family) identified as highly competent hosts for WNV [3]. In the urban or spillover cycle, the virus can be transmitted from birds to humans through bridge vectors, i.e. mosquito species that bite both birds and humans [4]. The virus has been detected in a plethora of mosquito species, but the most important for viral transmission are *Culex* species that feed on birds, including *Culex pipiens* [5].

In humans, the incubation period is usually 2–15 days [6]. Approximately 80% of those infected with WNV are asymptomatic and ca 20% develop symptoms, with varying severity [7]. West Nile fever (WNF) is the most common clinical manifestation, but < 1% of people develop West Nile neuroinvasive disease (WNND), when the virus affects the central nervous system, with or without imaging abnormalities [8]. Patients with WNND can develop encephalitis (ca 55–60%), meningitis (ca 35–40%) and acute flaccid paralysis (5–10%), while overlapping syndromes can also occur [8,9].

Seroprevalence studies provide a more accurate depiction of exposure to WNV, as they are not affected by limitations of clinical surveillance systems and can capture asymptomatic and oligosymptomatic infections which are rarely diagnosed. In those cases, the clinical manifestations, if present, are mild and non-specific and do not warrant further investigation. Therefore, seroprevalence studies can provide useful evidence on the actual infections and their distribution according to geographical and demographic characteristics. Usually, IgM antibodies specific to WNV are detected 3–8 days after the infection and last for 30–90 days, though longer duration has been recorded, with some cases retaining detectable levels of IgM antibodies for > 3 years [10,11]. Following a symptomatic or an asymptomatic infection, WNV IgG antibodies can be detected soon after IgM antibodies and remain for many years [11]. West Nile virus seropositivity can be determined for instance by ELISA. However, because WNV is antigenically close to other flaviviruses, cross-reactivity in ELISA is of concern [12,13].

The first human case of WNV infection was detected in Greece in 2010 [14], and since then, human cases have been recorded every year, except for 2015 and 2016. Greece has consistently ranked among the most affected countries in Europe [15]. Phylogenetic analyses on WNV genomic sequences obtained from infected mosquito pools indicate that WNV circulation in Greece is caused by WNV lineage 2 [16]. Since the appearance of WNV in Greece, two seroprevalence studies have been conducted. The first in the Regional Unit of Imathia, where the highest incidence of WNND was observed during an outbreak in 2010, and estimated a seroprevalence of 5.8% [17]. In a nationwide study between November 2012 and April 2013, the estimated national weighted seroprevalence for IgG antibodies was 1.55% (95% confidence interval (CI): 1.17–2.04) after plaque reduction neutralisation test (PRNT) [18]. Seropositivity was associated with age, while considerable spatial differences were seen. The estimated number of infected individuals per a case of WNND was 376, with the highest number among older adults.

We aimed to estimate WNV seropositivity in Greece, including spatial distribution. We also aimed to compare the results with the previous nationwide survey and to investigate the effect of possible demographic risk factors for WNV infection, such as sex and age.

## Methods

### Study design

The target population was the entire population residing in Greece during 2020. Samples were collected April–December 2020. To ensure comparability, we followed the same sampling plan as in the previous nationwide serosurvey. A geographically stratified sampling plan was applied to ensure a representative sample from all prefectures in Greece. Considering an expected seroprevalence of 4.5%, a precision of 2% and a 95% confidence level, 413 samples per Nomenclature of Territorial Units for Statistics level 1 (NUTS1) (https://ec.europa.eu/eurostat/web/nuts) region was deemed sufficient. The calculation was based on a formula (see below), where n is a sample size, z is z-score, p is the expected prevalence and d is the precision.

$n=\frac{z^{2}\;\times \;p\;\times \;q}{d^{2}}$

As the NUTS1 region of the Aegean Islands and Crete accounts for 10.5% of the total Greek population and sampling was proportional to population size, 4,000 samples were considered a sufficient sample size. Blood samples were also collected proportionally to five age categories in each stratum. In some NUTS3 regions, oversampling was considered appropriate (ca 1.5–2.5 times higher) due to discrepancies between seropositivity and WNND cases in the 2012–2013 survey. Thus, the sample size was larger than in the previous study consisting of 4,416 samples. To avoid biases due to disproportional sampling of some areas, all seroprevalence estimates were weighed by the regional unit (NUTS3) of residence (see Statistical analysis).

Serum samples were collected from public and private laboratories from all prefectures of Greece. Each laboratory provided a specified number of leftover samples corresponding to regional population demographics. The samples were taken from individuals visiting laboratory services for various purposes such as check-ups and chronic disease management.

### Surveillance data on West Nile virus infection

Nationwide data on cases of WNV and WNND for the study period were obtained from the National Public Health Organization (NPHO). The Vector-borne Diseases (VBD) Department of the NPHO coordinates a national active laboratory-based surveillance system which includes an in-depth investigation of every diagnosed WNV case. The disease is classified as WNND based on the clinical assessment by the treating physicians and/or additional laboratory and/or imaging findings, when available.

### Laboratory analysis

The serum samples were analysed at the Laboratory of Hygiene and Epidemiology of the University of Thessaly in Larissa, Greece. For WNV IgG determination, a commercially available ELISA kit, SERION ELISA classic West Nile Virus IgG (SERION Diagnostics, Virion\Serion GmbH, Würzburg, Germany) with the DYNEX DSX (DYNEX technologies, Chantilly, the United States (US)) fully automated ELISA processing system was used, following strictly the manufacturers’ specifications. The evaluation of optical measurement signals was performed automatically with the SERION easyANALYZE software (SERION Diagnostics). According to the software, specimens with an index value of ≥ 15.00 are characterised as positive for WNV IgG antibodies. An index value between 11.00 and < 15.00 was considered as equivocal, while an index value of < 11.00 was considered negative.

To examine whether the positive ELISA samples contained WNV-specific antibodies, virus neutralisation test (VNT) was conducted. In detail, WNV lineage 2 strain from the laboratory collection was cultured in vero cells CCL-81 from the American Type Culture Collection (ATCC, Manassas, US) using Eagle's Minimum Essential Medium (EMEM) (ATCC 30–2003) and 10% fetal bovine serum (FBS) (ATCC 30–2020) under biosafety level (BSL)3 conditions. The cells were incubated at 37°C for 4 days in 5% CO_2_ atmosphere.

All positive sera were twofold diluted (1:4–1:256) using EMEM. In a 96-well microplate (tissue culture, flat bottom) (TC plate 96, Sarstedt, Nümbrecht, Germany), 100 μL from each serum dilution and 100 μL of 50% tissue culture infectious dose (TCID50) cultured WNV were mixed, and the plate was incubated at 37°C for 60 min. Finally, a suspension of viable vero cells (1.5 × 104 cells in EMEM with 10% FBS and 2% antibiotic/antimycotic and 1% L-glutamine) was added to each well and further incubated at 37°C for 10 days in 5% CO_2_ atmosphere. Daily examination of the microplates using inverted microscope was conducted to observe for cytopathic effect (CPE). The serum dilution where no CPE was observed was recorded as the result of the VNT. In the statistical analysis, positive WNV IgG ELISA samples that were not subsequently confirmed by serum virus neutralisation were considered as negative.

### Statistical analysis

We performed the statistical analyses with R (https://www.r-project.org/), version 4.3.2. The packages ‘srvyr’ and ‘survey’ were used to calculate crude and adjusted seroprevalences. Adjusted seropositivity was calculated by weighting each observation according to age group, sex and regional unit. Weights were determined by the distribution of the population recorded by the Greek population census of 2011. Seroprevalence was expressed as a proportion (%) with the corresponding 95% CIs. The two proportion z-test was used to examine differences between seropositivity among deferent comparison groups (age groups, male/female, area of residence). For all analyses, a p value < 0.05 was considered statistically significant. Thematic maps were built with the use of the ‘tmap’ R package. The calculations for WNND incidence and the infected individuals per WNND case were based on case counts, population size and seroprevalence. Cumulative WNND incidence was determined by dividing the total number of cases to the total population, while the infected individuals per WNND case was calculated by dividing seroprevalence to incidence.

## Results

In total, 4,416 serum samples were analysed for the presence of IgG antibodies against WNV, and 138 (3.13%) samples were positive for WNV with ELISA. After virus neutralisation, 116 (2.63%) were confirmed. Supplementary Table 2 presents the ELISA titres and the results of the neutralisation tests.

The national weighted seroprevalence (adjusted for age group, region and sex) was 2.83% (95% CI: 2.32–3.44) (Table 1). The seropositivity rate was 3.06% in males and 2.62% in females, the difference was not statistically significant (p = 0.386). Seroprevalence increased with age, with the highest seropositivity in persons aged ≥ 80 years (6.04%; 95% CI: 3.28–10.88) and the lowest in those aged 25–54 years (1.92%; 95% CI: 1.36–2.69). No significant differences in seropositivity were seen between the four NUTS1 regions. The seropositivity rate was highest in Attica at 3.08% and lowest in Central Greece at 1.81%.

**Table 1:** Prevalence of IgG antibodies against West Nile virus, by age, sex and region, Greece, 2020 (n = 4,416)

Characteristics	ELISA IgG positives	NT positives	Samples tested (n)	Seropositivity (%)		95% CI	p value
				Crude	Adjusted		
Sex							
Female	74	63	2,590	2.43	2.62	1.99–3.43	0.386
Male	64	53	1,826	2.90	3.06	2.3–4.05	Reference
Age (years)							
≤ 24	30	25	1,224	2.04	2.42	1.6–3.64	Reference
25–54	50	37	1,816	2.04	1.92	1.36–2.69	1.000
55–64	18	17	578	2.94	3.35	2.02–5.5	0.311
65–79	25	24	597	4.02	4.22	2.71–6.51	0.022
≥ 80	15	13	201	6.47	6.04	3.28–10.88	0.001
NUTS1 region							
Attica	62	51	1,656	3.08	3.2	2.41–4.24	0.485
Central Greece	30	22	1,217	1.81	2.34	1.43–3.82	0.713
Northern Greece	35	34	1,146	2.97	2.88	2.02–4.10	0.580
Aegean Islands–Crete	11	9	397	2.27	2.51	1.21–5.12	Reference
Total	138	116	4,416	2.63	2.83	2.32–3.44	NA

CI: confidence interval; NA: not applicable; NT: neutralisation test; NUTS: Nomenclature of Territorial Units for Statistics.

The seroprevalence of the 2012–2013 and 2020 surveys and cumulative number of WNND cases per 1,000 inhabitants are presented (Figure). While the geographical distribution of cumulative WNND incidence indicates that some prefectures (East Macedonia and Thrace, Central Macedonia) were more affected, this was not confirmed by the serosurvey. Detailed information about the distribution at NUTS2 and NUTS3 level is provided in Supplementary Table 3.

**Figure:**
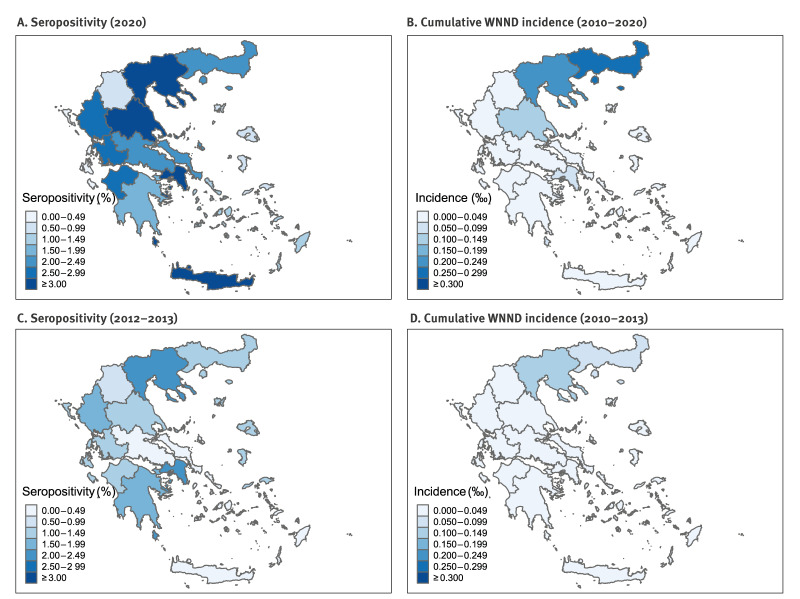
Maps presenting adjusted prevalence of IgG antibodies against West Nile virus and cumulative incidence of West Nile neuroinvasive disease, Greece, 2012–2013 (n = 3,962) and 2020 (n = 4,416)

NUTS: Nomenclature of Territorial Units for Statistics; WNDD: West Nile neuroinvasive disease.

The data are presented at NUTS level 2. Cumulative WWND incidence is presented as the total number of WWND cases per 1,000 inhabitants. Seropositivity is adjusted for age group and sex. A total of 973 WNND cases were notified 2010–2020.

We compared seropositivity of the 2012–2013 and 2020 serosurveys across NUTS1 regions (Table 2). Seropositivity increased between the two surveys, with a crude seropositivity rate of 1.54% in the 2012–2013 survey and 2.63% in the 2020 survey (p < 0.001). The increase in the seropositivity rates was seen throughout the country, with the highest increase in the Aegean Islands and Crete region, from 0.50% in 2012–2013 to 2.27% in 2020, although the increase was not statistically significant (p = 0.066).

**Table 2:** Comparison of crude prevalence of IgG antibodies against West Nile virus in surveys 2012–2013 (n = 3,962) and 2020 (n = 4,416), by geographical region, Greece

NUTS1	2012–2013			2020			p value
	Positive (n)	Samples (n)	%	Positive (n)	Samples (n)	%	
Attica	27	1,354	1.99	51	1,656	3.08	0.080
Central Greece	12	1,071	1.12	22	1,217	1.81	0.237
Northern Greece	20	1,136	1.76	34	1,146	2.97	0.078
Aegean Islands–Crete	2	401	0.50	9	397	2.27	0.066
Total	61	3,962	1.54	116	4,416	2.63	< 0.001

NUTS: Nomenclature of Territorial Units for Statistics.

We calculated the number of infections with WNV per notified case of WNND using the seroprevalence data of this survey and cumulative incidence 2010–2020 (Table 3). The overall number of infections per WNND case was 312 (95% CI: 256–379) which is consistent with the findings of the 2012–2013 survey (n = 376; 95% CI: 338–421).The highest number of infections per WNND case was observed in persons aged 0–24 years (n = 2,769; 95% CI: 2,117–4,529) while those aged ≥ 65 years had fewer infections per WNND case (65–79 years: n = 166; 95% CI: 102–246 and ≥ 80 years: n = 169; 95% CI: 114.6–342). Furthermore, the lowest number of infections per WNND case was seen in Northern Greece (n = 124; 95% CI: 85–180), while the highest number was seen in the Aegean Islands and Crete (n = 6,087; 95% CI: 2,939–12,429).

**Table 3:** Estimated number of persons infected with West Nile virus per a case of West Nile neuroinvasive disease, by age and region, Greece, 2010–2020

Characteristics	WNND cases	Population	Infections per WNND case	95% CI
Age (years)				
0–24	23	2,636,830	2,769	2,117–4,529
25–54	122	4,295,235	674	656–1181
55–64	125	1,400,300	375	229–622
65–79	427	1,612,960	166	102–246
≥ 80	276	773,240	169	114.6–342
NUTS1				
Attica	233	3,738,901	520	392–687
Central Greece	141	2,962,152	477	300–754
Northern Greece	588	2,803,980	124	85–180
Aegean Islands–Crete	5	1,213,532	6,087	2,939–12,429
Total	973	10,718,565	312	256–379

CI: confidence interval; NUTS: Nomenclature of Territorial Units for Statistics; WNND: West Nile neuroinvasive disease.

Infections per WNND case were calculated using adjusted seropositivity and cumulative incidence.

In seven NUTS3 regions, although seropositive samples were detected in the present survey, no WNND cases were recorded between 2010 and 2020. In these regions, the proportion of positive samples varied. Notably, in two of these areas (Messenia and Chania), seropositive samples were detected also in the 2012–2013 survey with no notified cases.

## Discussion

After adjusting for age group, sex and region, the seroprevalence was estimated at 2.83% which is an increase from 1.55% in the 2012–2013 survey [18]. Since the confirmation of the first human case of WNV infection in Greece, cases have been notified almost every year in Greece. The disease incidence increased 2018–2020, especially during the 2018 outbreak [19]. Our survey data indicate an increase in the exposure to WNV. Comparison with studies from other European countries indicates that Greece ranks among those with relatively high WNV seroprevalence rates. Recent studies report seroprevalences ranging from 1.2% in southern France [20], 1.5% in Bulgaria [21], 1.9% in northern Italy [22] to 3.2% in northwestern Romania [23] and 4.3% in Hungary [24].

We did not find a statistically significant difference in seropositivity between males and females, consistent with other studies [25]. However, a higher seroprevalence was identified in persons aged ≥ 80 years, and the risk appears to increase with age. The same pattern of increased seroprevalence in persons aged ≥ 65 years was observed in the 2012–2013 survey [18]. Similar observations have also been reported from other studies [23,26]. A possible explanation could be that rural areas in Greece, characterised by a higher risk of exposure to disease vectors, typically have a higher proportion of older residents. Other reasons could be spending time outdoors (e.g. during agricultural activities), not using measures to protect from mosquito bites and limited knowledge and education. As older adults are more susceptible to severe infection with neurological involvement, preventive public health measures are necessary.

Unlike the 2012–2013 study, no significant differences were observed between the NUTS1 regions, which indicates a spatial expansion of the virus in Greece. Rates of seropositivity increased in all NUTS1 areas. An unexpected finding was the discordance between regions with increased seroprevalence and those with increased incidence of WNND. We do not know where the seropositive persons had been exposed to the virus, in contrast to data on notified cases. The NPHO reports on WNV cases include an assumed location of exposure which is derived from patient interviews and communication with the treating physicians. The location of exposure may differ from the location of residence, and individuals who were tested positive for antibodies may have contracted the virus in different locations from their area of residence. According to the Hellenic Statistical Authority, 4.8 million individuals of all ages undertook at least one domestic trip, resulting in 8.3 million trips in 2022 [27]. Most of these travels (60.7%) took place during the summer months. Other possible explanations could be differences in the local surveillance systems. A discrepancy was observed between notified WNV cases and seroprevalence in certain NUTS2 and NUTS3 areas. Although the survey was designed to estimate seroprevalence at NUTS1 level, an additional finding of our study was an apparent viral circulation in regions with no history of reported cases like Messenia and Chania.

The significant geographical differences in the infections-to-WNND cases ratio suggest underdiagnosis in some regions. On the other hand, in some regions, the lower ratios maybe explained by increased awareness. For instance, in Northern Greece where the National Reference Centre for arboviruses operates, the ratio was the lowest (124; 95% CI: 85–180).

According to entomological surveillance data from Greece (2019–2020), *C. pipiens* is most abundant in Central Macedonia, followed by Thessaly and Attica regions [28]. In these regions, seroprevalences in the present study were marginally higher than the nationwide estimate. Additionally, we observed high seroprevalence in Crete and the Peloponnese, in contrast with case surveillance data. In a 2018–2020 study from Crete, *C. pipiens* was the dominant mosquito species in the western prefectures, with WNV antibodies detected in 1.7% of chicken serum samples [29]. The first human cases of WNND on the island of Crete were identified 2017–2018 [19]. In 2021, in the Aegean Islands and Crete region, a minimum infection rate (MIR) of mosquitoes was 12.05% in the South Aegean and 20.41% in the North Aegean areas [30]. Data from Crete, the most populated area in the region, was not included in the report. In the Peloponnese area, WNV RNA was detected in 46 wild birds of 10 species and in 22 mosquito pools [31].

The number of infections per WNND case also differed substantially with age groups, probably due to the very low incidence of WNND in younger individuals. Compared with the previous study, the overall ratio was similar (1:355 vs 1:312). In previous US studies, the ratios were lower, ranging from 1:140 [32] to 1:244 [33]. We assumed no seroreversion of IgG antibodies in the calculations of the ratios in our study. Although evidence indicates prolonged persistence of WNV antibodies over several years [11,34,35], the length of persistence is not clear. Thus, the ratios in our study may be higher if several infected individuals seroreverted between 2010 and 2020.

Some limitations of the present study should be considered when interpreting the results. The leftover sampling method is inexpensive, but the sampling framework consists of individuals undergoing haematological or biochemical tests. Additionally, we have no data on non-responders or non-participants to help us evaluate potential selection bias. However, this method has been used in seroepidemiological studies on vaccine-preventable diseases. Moreover, since we followed the same methodology as in the previous study, we could compare the results. As we did not collect metadata on factors like outdoor activity, urban or rural residence, travel to other WNV endemic areas, mosquito-repellent use and chronic diseases, interpretation of discrepancies between seroprevalence and WNND incidence was challenging. Finally, while our sample size was adequate to estimate seroprevalence at the national and NUTS1 levels with a reasonable margin of error, increasing the sample size would provide more precise estimates of seropositivity at the NUTS2 and NUTS3 levels and potentially uncover additional statistically significant associations.

## Conclusion

In conclusion, our study suggests a wider geographical spread of WNV infections in Greece compared with previous investigations and surveillance data based on clinical cases, although the virus circulation remained at relatively low levels. Our results indicate that serosurveillance provides essential evidence for public health action and policies. Serosurvey data can be integrated into the National Action Plans for WNV to refine risk assessment and intervention strategies.

## Supplementary Data

441000 Supplementary Material
