# Supplementary material for: Seroprevalence of West Nile virus, Greece, 2020
Source: Euro Surveill. 2025 Apr 17;30(15):2400487. doi: 10.2807/1560-7917.ES.2025.30.15.2400487 (PMC12007402; doi:10.2807/1560-7917.ES.2025.30.15.2400487)
Supplement: Supplementary Material [file 24-00487_HADJICHRISTODOULOU_Supplement.pdf]

This supplementary material is hosted by Eurosurveillance as supporting information alongside the article “Seroprevalence of West Nile virus , Greece, 2020” , on behalf of the authors, who remain responsible for the accuracy and appropriateness of the content. The same standards for ethics, copyright, attributions and permissions as for the article apply. Supplements are not edited by Eurosurveillance and the journal is not responsible for the maintenance of any links or email addresses provided therein.

Supplementary Table 1: Study population characteristics

**Factor**	N
	(N=4416)
**Age group**	
0-24	1224 (27.7%)
25-54	1816 (41.1%)
55-64	578 (13.1%)
65-79	597 (13.5%)
80++	201 (4.6%)
**Sex**	
Female	2590 (58.7%)
Male	1826 (41.3%)
**NUTS1**	
Aegean Islands-Crete	397 (9.0%)
Attica	1656 (37.5%)
Central Greece	1217 (27.6%)
Northern Greece	1146 (26.0%)

**Supplementary Table 2 :** ELISA titres and the results of the Serum Virus Neutralization for all positive samples			
**S/N**	**IgG Ab Elisa titre**	**VNT dilution***	**VNT caracterization**
1	37,53	1:256	Positive (+)
2	18,18	1:32	Positive (+)
3	29,81	1:128	Positive (+)
4	113,42	>1:256	Positive (+)
5	15,31	-	Negative (-)
6	17,06	-	Negative (-)
7	30,25	1:128	Positive (+)
8	15,16	-	Negative (-)
9	42,51	1:256	Positive (+)
10	48,89	1:256	Positive (+)
11	17,49	1:16	Positive (+)
12	19,59	1:64	Positive (+)
13	16,52	-	Negative (-)
14	15,58	-	Negative (-)
15	15,14	-	Negative (-)
16	46,29	1:256	Positive (+)
17	45,38	1:256	Positive (+)
18	34,62	1:128	Positive (+)
19	19,28	1:32	Positive (+)
20	57,2	>1:256	Positive (+)
21	33,81	1:128	Positive (+)
22	47,07	1:256	Positive (+)
23	68,14	>1:256	Positive (+)
24	25,36	1:64	Positive (+)
25	24,18	1:64	Positive (+)
26	31,05	1:64	Positive (+)
27	37,88	1:128	Positive (+)
28	24,37	1:64	Positive (+)
29	41,21	1:256	Positive (+)
30	44,52	>1:256	Positive (+)
31	21,12	1:32	Positive (+)
32	20,64	1:32	Positive (+)
33	20,76	1:32	Positive (+)
34	55,29	>1:256	Positive (+)
35	30,32	1:128	Positive (+)
36	15,58	-	Negative (-)
37	15,94	-	Negative (-)
38	16,09	-	Negative (-)
39	26,95	1:64	Positive (+)
40	17,59	-	Negative (-)

41	20,42	1:64	Positive (+)
42	17,46	1:32	Positive (+)
43	29,64	1:64	Positive (+)
44	30,99	1:128	Positive (+)
45	33,24	1:128	Positive (+)
46	19,57	1:32	Positive (+)
47	29,14	1:128	Positive (+)
48	26,31	1:64	Positive (+)
49	21,36	1:64	Positive (+)
50	16,56	-	Negative (-)
51	47,91	1:256	Positive (+)
52	28,41	1:128	Positive (+)
53	18,69	1:8	Positive (+)
54	21.36	1:64	Positive (+)
55	15,32	-	Negative (-)
56	15,66	-	Negative (-)
57	28.13	1:8	Positive (+)
58	15.03	1:4	Positive (+)
59	15.04	1:4	Positive (+)
60	25.33	1:4	Positive (+)
61	15.35	1:4	Positive (+)
62	21.16	1:16	Positive (+)
63	24.09	1:36	Positive (+)
64	17.25	1:4	Positive (+)
65	18.09	1:4	Positive (+)
66	19,31	1:16	Positive (+)
67	19.6	1:16	Positive (+)
68	30.02	1:8	Positive (+)
69	29.33	1:8	Positive (+)
70	22.71	1:16	Positive (+)
71	23.28	1:16	Positive (+)
72	23.62	1:64	Positive (+)
73	24.21	1:64	Positive (+)
74	26.25	1:16	Positive (+)
75	26.36	1:64	Positive (+)
76	27.23	1:64	Positive (+)
77	47.8	1:4	Positive (+)
78	37,29	1:4	Positive (+)
79	25.68	1:16	Positive (+)
80	50.77	1:4	Positive (+)
81	26,75	1:64	Positive (+)
82	20,75	1:16	Positive (+)
83	27,84	1:64	Positive (+)
84	21,52	1:16	Positive (+)

85	16.92	1:8	Positive (+)
86	29.22	1:8	Positive (+)
87	19,84	1:16	Positive (+)
88	26,79	1:64	Positive (+)
89	23,37	1:64	Positive (+)
90	49,01	1:8	Positive (+)
91	20,35	1:16	Positive (+)
92	24.41	1:64	Positive (+)
93	18.86	1:4	Positive (+)
94	29.03	1:8	Positive (+)
95	18.06	1:8	Positive (+)
96	21,61	1:8	Positive (+)
97	18.12	1:8	Positive (+)
98	18.47	1:16	Positive (+)
99	28.52	1:8	Positive (+)
100	21,87	1:64	Positive (+)
101	24,65	1:8	Positive (+)
102	30.94	1:8	Positive (+)
103	38,39	1:4	Positive (+)
104	15,03	-	Negative (-)
105	15,63	1:4	Positive (+)
106	19,6	1:64	Positive (+)
107	24,83	1:8	Positive (+)
108	21.95	1:64	Positive (+)
109	21,34	1:64	Positive (+)
110	24,57	1:8	Positive (+)
111	28.31	1:64	Positive (+)
112	31,72	1:8	Positive (+)
113	22.63	1:64	Positive (+)
114	19,39	1:16	Positive (+)
115	24,41	1:64	Positive (+)
116	16,05	1:4	Positive (+)
117	29,01	1:128	Positive (+)
118	17.23	1:8	Positive (+)
119	34,14	1:256	Positive (+)
120	16.3	1:8	Positive (+)
121	22.39	1:16	Positive (+)
122	22.5	1:16	Positive (+)
123	49,27	1:4	Positive (+)
124	19.77	1:8	Positive (+)
125	17,09	-	Negative (-)
126	19,32	1:32	Positive (+)
127	24.47	1:64	Positive (+)
128	51.15	1:4	Positive (+)
129	17.13	1:64	Positive (+)

130	32.71	1:8	Positive (+)
131	17.74	-	Negative (-)
132	16.44	-	Negative (-)
133	17.51	-	Negative (-)
134	15.41	-	Negative (-)
135	18,45	-	Negative (-)
136	19,23	1:8	Positive (+)
137	17.84	-	Negative (-)
138	18,21	-	Negative (-)

Supplementary Table 3 : Seroprevalence at NUTS 2 and NUTS 3 level

NUTS AREA	Code	NUTS LEVEL	Adj. Seroprevalence	Low CI	High CI	Total samples	VNT positive
Attiki	EL30	2	3,20	2,41	4,23	1656	51
Central Macedonia	EL52	2	3,09	2,02	4,70	737	23
Crete	EL43	2	3,33	1,42	7,61	225	6
Eastern Macedonia	EL51	2	2,19	1,07	4,44	304	8
and Thrace							
Ionian Islands	EL62	2	0,00	0,00	0,00	78	0
Epirus	EL54	2	2,94	0,88	9,35	105	3
North Aegean	EL41	2	0,97	0,13	6,88	72	1
Peloponnese	EL65	2	1,80	0,73	4,33	254	5
South Aegean	EL42	2	1,28	0,31	5,18	100	2
Central Greece	EL64	2	2,25	0,84	5,91	217	5
Thessaly	EL61	2	3,55	1,43	8,54	312	6
Western Greece	EL63	2	2,80	0,98	7,74	245	5
Western Macedonia	EL53	2	0,95	0,13	6,64	111	1
Attica	EL301-EL306	3	3,41	2,55	4,55	1453	48
Piraeus, Islands	EL307	3	1,78	0,51	6,02	203	3
Florina	EL533	3	5,25	0,61	33,46	20	1
Lesbos, Lemnos	EL411	3	0,97	0,13	6,88	72	1
Kalymnos, Karpathos, Kos, Rhodes (	EL421	3	1,28	0,31	5,18	100	2

former Dodekanisos Prefecture)							
Heraklion	EL431	3	1,94	0,48	7,54	124	2
Lasithi	EL432	3	3,61	0,46	23,41	35	1
Chania	EL434	3	5,95	1,66	19,16	66	3
Messenia*	EL653	3	5,64	2,27	13,36	97	5
Xanthi	EL512	3	0,00	0,00	0,00	52	0
Rhodope	EL513	3	3,40	0,81	13,19	69	2
Drama	EL514	3	5,89	2,03	15,86	68	4
Thasos, Kavala	EL515	3	2,71	0,64	10,77	53	2
Imathia	EL521	3	9,86	3,63	24,10	49	4
Thessaloniki	EL522	3	2,76	1,53	4,93	391	12
Kilkis	EL523	3	0,00	0,00	0,00	61	0
Pella	EL524	3	3,65	0,80	15,16	54	2
Pieria	EL525	3	3,15	0,73	12,56	47	2
Serres	EL526	3	1,33	0,18	9,44	64	1
Chalkidiki	EL527	3	2,30	0,55	9,05	71	2
Kozani*	EL531	3	0,00	0,00	0,00	58	0
Evros	EL511	3	0,00	0,00	0,00	62	0
Grevena*	EL531	3	0,00	0,00	0,00	14	0
Arta*	EL541	3	13,46	3,76	38,28	28	3
Kastoria	EL532	3	0,00	0,00	0,00	19	0
Thesprotia	EL542	3	0,00	0,00	0,00	17	0
Karditsa*	EL611	3	0,00	0,00	0,00	81	0
Trikala*	EL611	3	10,11	3,04	28,73	49	4
Larissa	EL612	3	2,45	0,58	9,84	107	2
Magnesia	EL613	3	0,00	0,00	0,00	75	0
Ioannina	EL543	3	0,00	0,00	0,00	60	0
Corfu	EL622	3	0,00	0,00	0,00	41	0
Aetolia-Acarnania	EL631	3	9,03	2,29	29,58	25	3
Achaea	EL632	3	0,00	0,00	0,00	126	0
Elis	EL633	3	1,54	0,37	6,15	94	2
Boeotia	EL641	3	0,00	0,00	0,00	48	0
Euboea	EL642	3	4,79	1,77	12,31	112	5
Ithaca, Cephaloni a	EL623	3	0,00	0,00	0,00	37	0
Phthiotis	EL644	3	0,00	0,00	0,00	27	0
Evrytania	EL643	3	0,00	0,00	0,00	12	0
Argolis*	EL651	3	0,00	0,00	0,00	35	0
Arcadia*	EL651	3	0,00	0,00	0,00	37	0
Korinthia	EL652	3	0,00	0,00	0,00	49	0
Phocis	EL645	3	0,00	0,00	0,00	18	0
Laconia*	EL653	3	0,00	0,00	0,00	36	0
